# Early exposure to flame retardants is prospectively associated with anxiety symptoms in adolescents: A prospective birth cohort study

**DOI:** 10.1002/da.23284

**Published:** 2022-10-11

**Authors:** Jeffrey R. Strawn, Yingying Xu, Kim M. Cecil, Jane Khoury, Mekibib Altaye, Joseph M. Braun, Bruce P. Lanphear, Andreas Sjodin, Aimin Chen, Kimberly Yolton

**Affiliations:** 1 Department of Psychiatry and Behavioral Neuroscience, Anxiety Disorders Research Program, College of Medicine, University of Cincinnati, Cincinnati, Ohio, USA; 2 Department of Pediatrics, Cincinnati Children’s Hospital Medical Center, Division of Clinical Pharmacology, Cincinnati, Ohio, USA; 3 Department of Pediatrics, Cincinnati Children’s Hospital Medical Center, Division of General and Community Pediatrics, Cincinnati, Ohio, USA; 4 Department of Radiology, Cincinnati Children’s Hospital Medical Center, University of Cincinnati College of Medicine, University of Cincinnati, Cincinnati, Ohio, USA; 5 Department of Environmental and Public Health Sciences, University of Cincinnati College of Medicine, University of Cincinnati, Cincinnati, Ohio, USA; 6 Department of Radiology, University of Cincinnati College of Medicine, University of Cincinnati, Cincinnati, Ohio, USA; 7 Department of Pediatrics, Division of Biostatistics and Epidemiology, Cincinnati Children’s Hospital Medical Center, Cincinnati, Ohio, USA; 8 Department of Epidemiology, Brown University School of Public Health, Providence, Rhode Island, USA; 9 BC Children’s Hospital Research Institute, Simon Fraser University, Burnaby, British Columbia, Canada; 10 Division of Laboratory Sciences, Centers for Disease Control and Prevention, Atlanta, Georgia, USA; 11 Department of Biostatistics, Epidemiology and Informatics, University of Pennsylvania Perelman School of Medicine, Philadelphia, Pennsylvania, USA

**Keywords:** anxiety, depression, polybrominated diphenyl ether (PBDE)

## Abstract

**Background:**

Anxiety disorders emerge during childhood and adolescence and are frequently preceded by subsyndromal anxiety symptoms. Environmental toxicants, including gestational polybrominated diphenyl ether (PBDE) exposure, are associated with neuropsychiatric sequelae; however, the role of PBDEs as risk factors for anxiety in adolescence is unclear.

**Methods:**

Using data from the Health Outcomes and Measures of the Environment (HOME) Study, a prospective pregnancy and birth cohort enrolled from 2003 to 2006, we investigated the relationship between gestational serum PBDE concentrations and anxiety symptoms in adolescents (*N* = 236). We measured five PBDE congeners (PBDE-28, −47, −99, −100, and −153) at 16 ± 3 weeks of gestation and calculated their sum (∑PBDE). We assessed self-reported anxiety symptoms using the Screen for Child Anxiety Related Emotional Disorders (SCARED) and depressive symptoms using the Children’s Depression Inventory (CDI-2) at age 12 years. We estimated the associations of maternal PBDE concentrations with child anxiety and depressive symptoms using multivariable linear regression and modified Poisson regression. Covariates included child sex, maternal race, maternal age at delivery, maternal marital status, maternal education, and household income at the 12-year study visit as well as maternal depressive and anxiety symptoms. Sensitivity analyses were performed to control for maternal lead and mercury at delivery.

**Results:**

After adjusting for predetermined covariates, each doubling in maternal PBDE concentrations was associated with increased SCARED scores (e.g., for ∑PBDE, SCARED total score, *β* = 1.6 95% confidence interval [CI]: 0.3-2.9, *p* = .019) and a nonsignificant increase in depressive symptoms (e.g., for CDI total score, *β* = .8, 95% CI: −0.2–1.8, *p* = .11).

**Conclusions:**

Gestational serum PBDE concentrations just before mid-pregnancy and during a period of active cortical and limbic neurogenesis, synaptogenesis and myelogenesis may be a risk factor for developing anxiety symptoms in early adolescence.

## 1 | INTRODUCTION

Anxiety disorders, which affect 7%–10% of children and adolescents, are the most common mental health conditions across the age span (Merikangas et al., 2010). They frequently emerge in late childhood and adolescence (Beesdo et al., 2010; Beesdo-Baum & Knappe, 2012) and are associated with significant impairment (Kendall et al., 2010; Ranøyen et al., 2018). Among the anxiety disorders, specific phobia and separation anxiety disorder (SAD) generally emerge prepubertally whereas generalized and social anxiety disorders as well as panic disorder and agoraphobia frequently emerge peri- or postpubertally (Beesdo et al., 2010; Beesdo-Baum & Knappe, 2012). Left untreated, anxiety disorders place children at high risk for developing mood disorders (Beesdo et al., 2010; Goodwin et al., 2004; Kinley et al., 2011; Lieb et al., 2016), substance misuse (Behrendt et al., 2011), suicidal behavior (Husky et al., 2012; Sareen et al., 2005), and economic disadvantage in adulthood (Asselmann et al., 2018).

Many psychological, biological and developmental risk factors increase the risk of developing anxiety (Strawn et al., 2021; Walkup et al., 2022). These include having an anxious parent (Beesdo et al., 2010), having specific personality disorder symptoms (Steinsbekk et al., 2019), family environment (Beesdo et al., 2010), early separation or institutionalization (Ellis et al., 2004) and specific attachment styles (Warren et al., 1997) and temperaments. Further, cognitive factors increase the risk of developing anxiety and anxiety disorders in youths and include threat bias, intolerance of uncertainty (Yook et al., 2010) and learned behaviors (e.g., avoidance) (Lau & Waters, 2017).

Exposure to environmental toxicants may also increase the risk of developing anxiety or anxiety disorders. In a longitudinal study of nearly 320 mothers in the Health Outcomes and Measures of the Environment (HOME) Study, maternal mercury concentrations during pregnancy were associated with more parent-reported anxiety in their children at age 8 years (Patel et al., 2019). Additionally, traffic related air pollution may increase generalized anxiety symptoms (Yolton et al., 2019) and, interestingly, this association may be mediated by myo-inositol concentrations in the cingulate cortex (Brunst et al., 2019). Further, specific components of air pollution that induce inflammation and oxidative stress (e.g., particulate matter <2.5 μm [PM_2.5_]) have been linked to exacerbations of anxiety in youths (Brokamp et al., 2019).

While accumulating data in children and adolescents suggest that environmental toxicants increase the acute and incipient risk of developing anxiety, few studies have investigated exposure to toxicants as early risk factors. One class of environmental toxicants that is particularly relevant to the development of anxiety is polybrominated diphenyl ethers (PBDEs), a class of chemicals widely used as flame retardant additives in polyurethane foams, furniture, carpet padding, car seats, and electronics. PBDEs are not covalently bound to product materials and, as such, are released from material surfaces during regular use. PBDEs are ubiquitous and highly lipophilic; thus, they readily cross the placenta, accumulate in human tissue and can persist in the body for up to a decade (Thuresson et al., 2006). PBDEs have been phased out in the United States, yet products containing them are still present in homes and offices worldwide (Al-Omran et al., 2021; Bennett et al., 2015; Dodson et al., 2012; Mitro et al., 2016) and meta-analyses of US studies examining PBDEs in indoor dust suggest that these compounds remain present (Mitro et al., 2016). Further, the PBDE exposures relevant for the maternal exposure in this study continue to pose a threat despite being banned in 2004. There is no recall of products such as furniture and, beyond this, products are donated and sold to other families, so PBDE exposure continues despite being banned from production.

PBDE exposure during early brain development is associated with cognitive deficits (Azar et al., 2021; Ding et al., 2015; Eskenazi et al., 2013; Herbstman et al., 2010) in language development (Ding et al., 2015), reading ability (Liang et al., 2019), working memory and executive function (Braun et al., 2017; Vuong et al., 2018) (Supporting Information: Table 1). In Chinese toddlers (24 months of age, *N* = 149), cord blood concentrations of PBDEs were associated with reduced language skills (sentence structure, semantics, vocabulary, comprehension) (Ding et al., 2015). Additionally, in the HOME Study, a prospective pregnancy and birth cohort, greater early childhood exposure to PBDEs (age 1, 2, 3, 5, and 8 years) was associated with decreased reading skills at age 5 and 8 years and decreased full scale intelligence quotient scores (Liang et al., 2019) as well as decreased behavioral regulation and emotional control at age 8 years (Vuong et al., 2018). Further, PBDEs have been associated with ADHD symptoms (Chen et al., 2014; Cowell et al., 2015; Sagiv et al., 2015). For example, in another birth cohort study, prenatal exposure to PBDEs was associated with poorer attention and executive function, based on both parent report and neuropsychological testing in children aged 9 and 12 years (Sagiv et al., 2015). In other cohorts, cord PBDE concentrations have been associated with inattentive symptoms on the Child Behavior Checklist (CBCL) through age 7 years (Cowell et al., 2015). However, PBDEs have not been explored in relation to anxiety, particularly during early adolescence–a critical period when anxiety disorders frequently emerge (Wehry et al., 2015). We sought to examine the relationship between gestational exposure to PBDEs and the development of anxiety symptoms in young adolescents. Young adolescents were selected given that epidemiologic studies consistently reveal that anxiety symptoms are emerging during this period and that most prior studies of PBDE associations with behavioral disorders are in school-aged children with few studies extending these associations with anxiety or depressive symptoms in adolescence. We hypothesized that gestational PBDE exposure would increase the risk of anxiety symptoms in early adolescence.

## 2 | METHODS

### 2.1 | Study participants

From 2003 to 2006, the HOME Study, an ongoing prospective pregnancy and birth cohort, enrolled 468 pregnant women at 16 ± 3 weeks of gestation from 9 prenatal clinics in the greater Cincinnati, Ohio area. Details regarding inclusion and exclusion criteria, measurement of chemicals, and neurobehavioral assessments can be found elsewhere (Braun et al., 2017, Braun, Buckley et al., 2020). A total of 420 children (11 twins included) completed at least one follow-up visit between birth to age 12 years and 256 completed the 12-year study visit. Of the 256, 243 adolescents (7 twins included) for whom maternal serum PBDEs had been measured during pregnancy completed the 12-year assessment. We further randomly excluded 1 child from each twin set, resulting in a final sample size of 236 adolescents for the present study. The institutional review board at the Cincinnati Children’s Hospital Medical Center approved this study and, mothers and adolescents provided written informed consent and assent, respectively. Characterization of the participants and their mothers has been previously described (Braun et al., 2017).

### 2.2 | Internalizing behaviors assessment

At the 12-year study visit, participants completed the Screen for Child Anxiety Related Emotional Disorders (SCARED), a self-report measure developed to screen youth for anxiety disorders (Birmaher et al., 1997). The SCARED has been evaluated in outpatient mental health clinics (Desousa et al., 2013), randomized, prospective treatment studies (Caporino et al., 2017), and community-based populations. The SCARED consists of 41 questions and responses are rated on a scale of 0 to 2. The SCARED can be used to yield a total score as well as five subscale scores: generalized anxiety disorder (GAD), social phobia/social anxiety, SAD, somatic symptoms/panic disorder, and school phobia. In general, total SCARED scores ≥25 produce optimal cutoffs for clinically concerning symptoms. Additionally, cutoff values for clinically relevant scores in SCARED were examined (panic/somatic (≥7), generalized anxiety (≥9), separation anxiety (≥5), social anxiety (≥8), and school avoidance (≥3).

To explore the specificity of the relationship between PBDE exposure and anxiety, we also examined participant-reported depressive symptoms, using the Children’s Depression Inventory, 2nd Edition (CDI-2), a 27-item self-report inventory developed by Kovacs and Beck (1977) to measure depressive symptoms and affect in children and adolescents. Items are presented as three statements of varying symptom severity, and *T*-scores are generated based on normative data for males and females from 7 to 16 years of age.

### 2.3 | Measures of potential covariates

We considered the following as potential covariates in our analysis based on prior studies demonstrating their relationship with internalizing outcomes or their potential role as a confounder in the relationship between PBDE exposure and anxiety/depressive symptoms: child sex, maternal race, maternal age at delivery, maternal marital status, maternal education, and household income at the 12-year study visit, as well as the relationship frustration score from the Behavioral Assessment System-3 Parenting Relationship Questionnaire (BASC-3 PRQ). Mothers completed the Symptom Checklist-90-Revised (SCL) (Derogatis, 1994), a multidimensional self-report that assesses psychiatric symptoms across nine dimensions (e.g., somatization, obsessive compulsion, interpersonal anxiety, depression, anxiety, etc.,). To control for the effects of maternal depressive or anxiety symptoms, the maternal SCL anxiety score was considered as a potential covariate for SCARED outcomes, and the maternal SCL depression score for CDI outcomes. Finally, given that maternal lead and mercury (Patel et al., 2019) have been associated with anxiety, we also considered maternal whole blood lead and total mercury measured during pregnancy in secondary analyses. Quantification of maternal blood lead and mercury concentrations in this sample has been described previously (Braun, Yolton, et al., 2021; Patel et al., 2019).

### 2.4 | PBDE exposure determination

Whole blood was collected from pregnant women at approximately 16 weeks gestation, and for two women at ~26 weeks gestation. Sera were separated and stored at −80°C until measurement of PBDE congeners (−28, −47, −99, −100, and −153) using gas chromatography/isotope dilution high-resolution mass spectrometry (Sjödin et al., 2004). Details about PBDE measurements, including quality assurance, imputation of measurements below the limit of detection, and lipid standardization, have been previously described (Vuong et al., 2017). Briefly, PBDE concentrations that were less than the detection limit, were substituted with detection $\mathrm{limit}/\sqrt{2}$ Hornung and Reed (1990). Serum PBDE concentrations were standardized by serum total lipid concentrations to account for the lipophilic nature of these compounds and their diurnal variation (O’Brien et al., 2016; Phillips et al., 1989).

### 2.5 | Statistical analyses

Descriptive statistics were used to summarize and examine the data distribution of adolescent, maternal, and household characteristics at the 12-year visit and identify potential outliers across exposure and outcome measures. Means and standard deviations or medians and interquartile ranges are reported for the continuous variables, as appropriate; frequencies and percentages are reported for categorical variables. We log_2_-transformed the PBDE concentrations before further statistical analysis to reduce variation and the influence of extreme values.

Linear regression models were used to assess the association between maternal PBDE concentrations (each congener and total PBDEs which is the sum of 5 individual congeners [−28, −47, −99, −100, −153], examined separately) and SCARED scores as well as between PBDE concentrations and CDI-2 scores. We examined unadjusted models first and then developed covariate-adjusted models. Guided by a directed acyclic graph of the hypothesized causal diagram for our analysis (Supporting Information: Figure 1), we included the following covariates in the adjusted models: child sex (for SCARED outcomes only as CDI-2 *T*-scores are already adjusted for sex), maternal age, marital status, education, income at 12-year visit, maternal SCL-90 anxiety score (for SCARED outcomes), maternal SCL-90 depression score (for CDI-2 outcomes), and PRQ relational frustration score. As a secondary analysis, we additionally adjusted for maternal blood lead and mercury. We performed two sensitivity analyses (1) excluding 3–7 participants with extreme values in PBDEs (*n* = 5, 6, 6, 6, 7, 3 for analysis of PBDE28, 47, 99, 100, 153, and ∑PBDE, respectively); (2) including only singletons (*n* = 229). We evaluated potential effect measure modification of the child’s sex by including a PBDE-by-sex interaction term in the regression models; this interaction was considered to be statistically significant if its *p* < 0.1. We also examined the association between PBDE concentrations and a categorized SCARED total or subscale score in the clinically significant range (e.g., total score ≥25, panic score ≥7, generalized anxiety ≥9). We used modified Poisson regression models to estimate the relative risks (RRs) (Zou, 2004), adjusting for the same covariates as the linear regression models. Last, a post hoc analysis was performed to examine the PBDE-by-sex interaction effect for SCARED and CDI scores. Statistical analyses were performed using SAS^®^ version 9.4 (SAS Institute).

## 3 | RESULTS

### 3.1 | Characteristics of participants

The 236 adolescents included in the present study were on average age 12 years (12.4 ± 0.7) at the time of the study visit; 55.9% were female; mothers were on average age 29 years at delivery (29.3 ± 5.7 years). There was a diverse range of household income levels, with the median income being $75,000 (interquartile range: $35,000-$145,000) at the 12-year visit. The majority of participants (79%) were Tanner Stage 3–5; 24 (18.5%) and 3 (2.3%) were Tanner Stages 2 and 1, respectively. Similarly, 51% of males were Tanner Stage 3–5 while 33 (32%) and 18 (17%) were Tanner Stage 2 and 1 respectively. Additional characteristics of the study participants and their mothers are shown in Table 1. In general, characteristics of the adolescents did not vary between those included in the analysis and those not included (*n* = 184) either because they did not complete the 12-year study visit (*n* = 164), did not have complete data available for the analysis (*n* = 13), or among the excluded twins (*n* = 7) (Table 1). Of the 236 participants included in the present study, 9 reported antidepressant treatment at either the 8-year visit or 12-year visit (citalopram, *n* = 2; escitalopram, *n* = 2; fluoxetine, *n* = 2; sertraline, *n* = 2); however, medical record-recorded diagnoses of anxiety disorders were not included.

### 3.2 | Relationship between PBDE and anxiety symptoms

The five PBDE congeners were detected in most of participants (87% to 99.2%); PBDE-47 was the most abundant congener (geometric mean: 20.8 ng/g lipid) (Supporting Information: Table 2). Total SCARED scores ranged from 0 to 65 (*N* = 236, mean: 21.1 ± 12.5) and were higher in females (23.2 ± 13.7) compared to males (18.4 ± 10.4). The average score was 4.9 ± 4.2 for panic symptoms (range: 0–21), 5.0 ± 3.7 for generalized anxiety (range: 0–16), 4.2 ± 3.1 for separation anxiety (range: 0–15), 5.4 ± 3.3 for social anxiety (range: 0–14) and 1.8 ± 1.6 for school avoidance (range: 0–8). Twenty-eight percent of the participants had scores above the clinically significant threshold for panic symptoms, 17% had scores that exceeded the clinically significant threshold for generalized anxiety. For separation anxiety, social anxiety, and school avoidance, 38%, 24%, 28% exceeded clinically significant thresholds respectively. Finally, 35% of the adolescents had scores above the clinically significant cutoff in total score.

**FIGURE 1:**
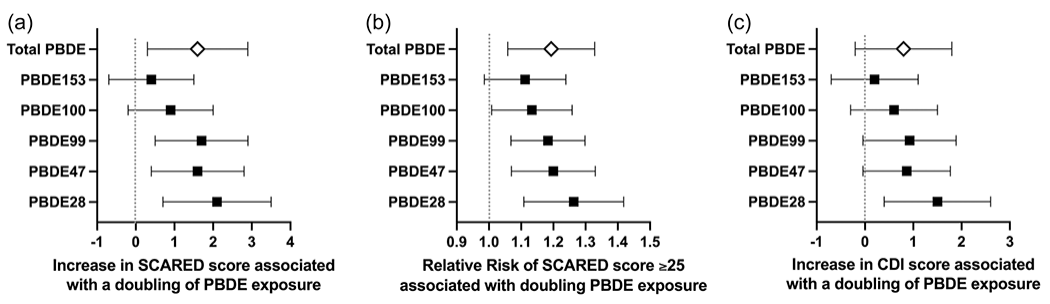
Maternal serum polybrominated diphenyl ether concentrations and Internalizing Symptoms at 12 Years. Anxiety (a, b) and depressive (c) symptoms are reflected by the total scores on the child-reported Screen for Child Anxiety and Related Emotional Disorders (SCARED) scores and the Children’s Depression Inventory (CDI). Analysis of anxiety symptoms is adjusted for child sex, maternal age, marital status, education, income at 12-year visit, maternal Symptom Checklist-90-R anxiety score, and relational frustration score while analysis of depressive symptoms is adjusted for maternal age, marital status, education, income at 12-year visit, relational frustration score, and maternal Symptom Checklist-90-R depression score.

With adjustment for covariates, higher maternal serum PBDE concentrations were consistently associated with increased anxiety symptoms (Table 2). For example, increases in SCARED total scores corresponding to each doubling of PBDE concentrations were: 2.1 (95% confidence interval [CI]: 0.7–3.5, *p* = .004) for PBDE-28, 1.6 (95% CI: 0.4–2.8, *p* = .008) for PBDE-47, 1.7 (95% CI: 0.5–2.9, *p* = .004) for PBDE-99, and 1.6 (95% CI: 0.3–2.9, *p* = .0019 for ∑PBDE. Positive associations were also observed between anxiety symptoms and PBDE-100 (*β* = .9, 95% CI: −0.2–2.0, *p* = .116) or PBDE-153 (*β* = .4, 95% CI: −0.7–1.5, *p* = .451); however, the 95% CI included the null value (Figure 1).

Higher PBDE concentrations were consistently associated with an increased RR of a clinically-relevant SCARED total score (≥25): for PBDE-28 (RR for each doubling of PBDE concentration: 1.26, 95% CI: 1.11–1.42, *p* < .001), PBDE-47 (RR: 1.20, 95% CI: 1.07–1.33, *p* < .001), PBDE-99 (RR: 1.18, 95% CI: 1.07–1.30, *p* = .001), PBDE-100 (RR: 1.13, 95% CI: 1.01–1.26, *p* = .027), PBDE-153 (RR: 1.11, 95% CI: 0.99–1.24, *p* = .082) as well as ∑PBDE (RR: 1.19, 95% CI: 1.06–1.33, *p* = .002) (Figure 1).

Additionally, higher PBDE-28, PBDE-47, PBDE-99 as well as ∑PBDE concentrations were associated with increased RR of an elevated panic and separation anxiety score above the clinical threshold (Table 3) whereas higher PBDE-100 concentrations were associated with increased RR of an elevated panic score above the threshold (Table 3).

In sensitivity analyses, we found that excluding adolescents with extremely high PBDE concentrations or excluding all twins yielded similar results (results not shown). Additionally adjusting models for maternal blood mercury concentrations and blood lead concentrations (Supporting Information: Table 3) did not meaningfully change these relationships. We did not find evidence of effect measure modification by child sex for all PBDEs congeners as well as ∑PBDE (*p* values for the sex-by-PBDE interaction ranged from 0.12 to 0.62).

### 3.3 | Relationship between PBDE and depressive symptoms

The total CDI-2 *T*-scores ranged from 40 to 90 (*N* = 236, mean: 51.9 ± 10.3) and were similar for females (53.1 ± 11.1) and males (50.4 ± 9.0). The average *T*-score was 51.7 ± 9.8 for emotional problems, 52.7 ± 10.9 for negative mood, 49.4 ± 8.7 for negative self-esteem, 51.6 ± 10.6 for functional problems, 52.5 ± 10.5 for ineffectiveness and 47.5 ± 10.0 for interpersonal problems. Each doubling of PBDE-28 concentration was associated with increases in total CDI-2 *T*-scores (*β*: 1.5, 95% CI: 0.4–2.6, *p* = .007) in addition to emotional problems (*β*: 1.2, 95% CI: 0.2–2.3, *p* = .021), negative mood (*β*: 1.3, 95% CI: 0.2–2.5, *p* = .028), functional problems (*β*: 1.6, 95% CI: 0.5–2.7, *p* = .006) and ineffectiveness (*β*: 1.8, 95% CI: 0.7–2.9, *p* = .002). Higher PBDE-99 concentrations were associated with increased negative mood *T*-scores (*β*: 1.1, 95% CI: 0.1–2.1, *p* = .038). However, other PBDE congeners and ∑PBDE were not significantly associated with total CDI-2 *T*-scores (Figure 1) or the *T*-scores for individual domains (Table 4), although they exhibited a similar directionality and magnitude of effect.

### 3.4 | The impact of biological sex on the relationship between PBDE and depressive symptoms

A sex-stratified analysis of the total SCARED scores and total CDI scores (Supporting Information: Table 4) did not reveal any statistically significant PBDE-by-sex interactions for specific congeners or total PBDE. However, there were more significant associations among males than females for both SCARED and CDI scores. In general, the directionality of effect was similar in males and females, although the analysis was not powered to detect sex differences in the effect of PBDE on SCARED or CDI scores.

## 4 | DISCUSSION

To our knowledge, this is the first study to examine the effects of in utero PBDE exposure on the development of anxiety symptoms in adolescence. Our findings suggest that the gestational effects of PBDE exposure extend beyond cognitive deficits and ADHD-related symptoms to also increase the risk of developing significant anxiety symptoms. In our sample, adolescents with higher gestational PBDE concentrations experienced more anxiety–and to a lesser degree with depressive symptoms. These findings have important clinical implications for primary and secondary prevention and for our understanding of the pathophysiology of anxiety disorders.

The development of anxiety disorders may relate to maturational changes in the structure, function and functional connectivity of multiple prefrontal cortical structures (Jarcho et al., 2015). Prior work suggests that neurostructural cortical changes are associated with anxiety disorders (Gold et al., 2017; Strawn et al., 2014) and may relate to developmental processes that occur in utero (e.g., synaptic density). It remains to be determined whether PBDE exposure relates to cortical changes observed in anxious adolescents, as suggested by in vitro studies wherein PBDE exposure inhibits neurite outgrowth in a dose-dependent manner (Bradner et al., 2013). However, one study involving children revealed that prenatal PBDE exposure alters intrinsic functional network organization (de Water et al., 2019). As such, PBDE-related effects on the developing brain could produce a cortex that is primed to support the subsequent development of anxiety.

Importantly, in preclinical models, early PBDE exposure alters molecular and neuronal signaling cascades (e.g., protein kinase C) (Kodavanti & Ward, 2005) and affects GABAergic and glutamatergic neurotransmitter systems, particularly within the prefrontal cortex (Bradner et al., 2013), Accumulating data also suggest PBDEs affect cholinergic transmission, induce oxidative stress (Bellés et al., 2010), modify DNA methylation (Woods et al., 2012), and alter brain proteins expression (e.g., CaMKII, GAP-43, synaptophysin, and tau) as well as NMDA receptor expression (Buratovic et al., 2014). Given the ubiquity of cholinergic, glutamatergic and GABAergic transmission as well as their developmental importance, these neurochemical and molecular effects of PBDEs during this developmental window could set the prime functional and structural fear circuitry for the development of anxiety in adolescence. Additionally, PBDE was measured just before mid-pregnancy, at 16 weeks gestation–a period that coincides with active neurogenesis within the neocortex, amygdala, limbic cortex, and striatum as well as completion of hippocampal subfield development and the early phases of synaptogenesis, myelogenesis and gliogenesis (Bayer et al., 1993; Xu et al., 2021; Zhang et al., 2013).

We found that prenatal PBDE exposure was most consistently associated with panic and social anxiety symptoms. Several lines of evidence suggest that social anxiety in children and young adolescents increases the risk for developing other disorders, including tripling the risk of developing GAD, more than doubling the risk of developing a mood disorder, and quadrupling the risk of developing panic disorder (Lieb et al., 2016). Similarly, having panic attack symptoms earlier in childhood or adolescence has been associated with an increased risk of developing other anxiety and depressive disorders (Asselmann et al., 2014; Pine et al., 1998; Wolitzky-Taylor et al., 2014). Further, youths with core features of an anxiety disorder or who may have subthreshold symptoms, as in this sample, are at risk of subsequently developing these disorders (Wolitzky-Taylor et al., 2014). Data from pre-adolescents suggest that those who endorsed core features of anxiety or had “subthreshold or subclinical manifestations” were more likely to meet diagnostic criteria for an anxiety disorder and the accompanying functional impairment and distress later in adolescence (Wolitzky-Taylor et al., 2014). Thus, the risk of increased anxiety symptoms in the clinically significant range associated with PBDE exposure highlights the need to further reduce environmental exposures to these compounds which might not only lower the risk of developing syndromic anxiety disorders and mood disorders. Additionally, this is especially important in early adolescence, a period during which the risk of developing anxiety disorders increases exponentially (Beesdo et al., 2010).

The persistence of PBDEs in the environment and their tardive effects on externalizing and internalizing symptoms highlights the need to better understand their neurotoxicity, particularly with regard to the potential mechanisms of these effects on prefrontal circuitry that is functionally implicated in the pathogenesis of anxiety disorders (Strawn et al., 2012, 2021). Additionally, it remains possible that the neurotoxic effects of PBDEs have differential and specific effects during critical developmental windows. For example, gestational PBDE exposure and contemporaneous subcortical development may give rise to symptoms while later exposure may differentially affect processing or even other internalizing symptoms (e.g., depression). Thus, these findings instantiate the need to clarify how the timing of exposures affects outcomes and to identify prenatal and postnatal periods of vulnerability during which PBDE-exposure produces specific or persistent neurodevelopmental changes. Ultimately, understanding how timing of exposure affects subsequent symptoms could provide better understanding of the interaction of environmental risk factors with intrinsic (e.g., genetic, psychological) risk factors for anxiety disorders.

### 4.1 | Limitations

This study has several limitations. First, our study focuses on a specific time period–early adolescence. Importantly, while anxiety symptoms may begin earlier, this is the time period of maximal increases in anxiety symptoms and the emergence of anxiety disorders. Thus, it remains possible that, because of disease progression or later emergence of symptoms, additional developmental effects could accentuate or dampen the magnitude of the PBDE associations observed herein. Second, self-report measures have inherent limitations (Walkup et al., 2022) and may differ from symptoms assessed by clinicians. Third, whether these symptoms represent psychiatric disorders (e.g., GAD, social anxiety disorder) cannot be determined in this sample as we did not include a clinician assessment. As such, despite the sensitivity for SCARED scores with regard to diagnostic cutoffs based on clinical global impression-severity scores and diagnostic confirmation (Caporino et al., 2017) in the Child and Adolescent Anxiety Multimodal Study (CAMS), the SCARED is primarily a screening tool and limits our ability to draw any conclusive associations between exposure risk and disease development. Fourth, while including lead and mercury in our models did not change the magnitude of the associations, environmental exposures, other than those examined herein could contribute to the risk of developing anxiety symptoms. Fifth, we examined anxiety symptoms at 12 years of age and do not have SCARED scores at earlier or later time points. We specifically chose early adolescence given this is a developmental period in which internalizing disorders often emerge. As this is the first study to identify associations between PBDEs and anxiety symptoms, there is no prior evidence suggesting that PBDEs-associated anxiety would have a different trajectory of symptoms or need for intervention than anxiety resulting from other causes. Additional research will be necessary to understand the persistence of these symptoms. We are currently conducting follow-up studies with our participants and hope to shed more light on this matter in a few years. Additionally, our assessment at age 12 precludes assessing the longitudinal stability, progression, or continuity of specific symptoms and the examination of anxiety disorder emergence.” Sixth, we evaluated PBDE exposure at 16 weeks given that we hypothesized that brain maturation processes would be more sensitive to PBDE exposure; however, as with any epidemiologic study, there may be periods of even greater susceptibility during pregnancy, particularly given the long half-life of PBDEs and high within-individual correlation. Finally, as with most single site studies, there are inherent limitations regarding the degree to which findings can be generalized to a larger (e.g., national) sample. Specifically, Hispanic, Asian Pacific and Indigenous American mothers were not well represented and the way in which PBDE exposure relates to internalizing symptoms in these populations may be difficult to generalize from the current study. Also, regarding generalizability, mothers in our cohort had modestly lower PBDE concentrations than pregnant women in the National Health and Nutrition Examination Survey (NHANES) examined around the same time (Derogatis, 1994).

## 5 | CONCLUSION

We found that gestational PBDE concentrations are significantly associated with increased anxiety symptoms in young adolescents. Moreover, the effects may be stronger for specific anxiety symptoms, including panic and separation anxiety. Due to the environmental persistence of PBDEs and ubiquitous exposure (Al-Omran et al., 2021; Dodson et al., 2012), more research is needed to understand the mechanisms by which exposure produce these effects and whether these associations persist later into adolescence.

## Supplementary Material

Supplemental

## Figures and Tables

**TABLE 1:** Cohort characteristics of adolescents included in current study and those not included

Characteristic	Included (*n* = 236)	Not included (*n* = 184)
Child sex, female	132 (55.9%)	94 (51.1%)

Maternal age at delivery (mean, SD)	29.3 ± 5.7	29.3 ± 5.8

Maternal race/ethnicity		
Non-Hispanic Black	83 (35.2%)	48 (26.8%)
Non-Hispanic White	141 (59.7%)	116 (63%)
Hispanic	4 (1.7%)	5 (2.7%)
Asian Pacific	5 (2.1%)	5 (2.7%)
Indigenous American	3 (1.3%)	5 (2.7%)
Unknown		5 (2.7%)

Maternal marital status at baseline^a^		
Married	148 (65.2%)	121 (67.6%)
Not married, living with partner	25 (11.0%)	31 (17.3%)
Not married, living alone	54 (23.8%)	27 (15.1%)

Maternal marital status at 12-year visit		
Married	155 (65.7%)	
Not married, living with partner	23 (9.7%)	
Not married, living alone	58 (24.6%)	

Maternal education at baseline		
High school or less	49 (21.6%)	46 (25.7%)
Some college	66 (29.1%)	36 (20.1%)
College graduate	70 (30.8%)	50 (27.9%)
Graduate or professional	42 (18.5%)	47 (26.3%)

Maternal education at 12-year visit		
High school or less	33 (14.0%)	
Some college	77 (32.6%)	
College graduate	68 (28.8%)	
Graduate or professional	58 (24.6%)	

Household income at baseline (Median [25th %ile, 75th %ile])	$55 K ($27.5 K, $85 K)	$55 K ($22.5 K, $85 K)

Household income at 12-year visit (Median [25th %ile, 75th %ile])	$75 K ($35 K, $145 K)	

Maternal BDI-II score at baseline (mean, SD)	9.9 ± 6.6	10 ± 7.2

Maternal BDI-II score at 12-year visit (mean, SD)	6.7 ± 6.7	

Maternal IQ (mean ± SD)	106 ± 14.7	106 ± 14.6

BASC-3 PRQ relation frustration score (mean ± SD)^b^	48.1 ± 9.1	48.5 ± 9.8

Maternal SCL depression score (mean ± SD)^b^	49.9 ± 9	50.1 ± 10.1

Maternal SCL anxiety score (mean ± SD)^b^	44.6 ± 8.8	45.2 ± 9

Abbreviations: BDI-II, Beck Depression Inventory-II; BASC PRQ, Behavioral Assessment System for Children, third edition, Parenting Relationship Questionnaire; SCL, symptom checklist-90-R.

a Comparison between children included and those not included significantly different (*p* > .05).

b Among those not included, PRQ scores were compared from either the 8 or 12-year visit; SCL scores were compared from either the 4–5-, 8-, or 12-year visit.

**TABLE 2:** Difference in scores of Screen for Child Anxiety and Related Disorders (SCARED) by each doubling of maternal serum PBDE concentration (ng/g lipid)

		Unadjusted		Adjusted^a^	
Congener	SCARED score	*β* estimate (95% CI) for log_2_ PBDE concentration	*p* Value	*β* estimate (95% CI) for log_2_ PBDE concentration	*p* Value
PBDE-28	Total score	2.5 (1, 3.9)	.001	2.1 (0.7, 3.5)	.004
	Panic	0.9 (0.4, 1.4)	<.001	0.8 (0.3, 1.2)	.002
	Generalized anxiety	0.4 (−0.03, 0.8)	.070	0.3 (−0.1, 0.8)	.132
	Separation anxiety	0.5 (0.2, 0.9)	.005	0.4 (0.1, 0.8)	.018
	Social anxiety	0.4 (0.04, 0.8)	.031	0.3 (−0.03, 0.7)	.072
	School avoidance	0.2 (0.04, 0.4)	.017	0.2 (0.02, 0.4)	.029

PBDE-47	Total score	2 (0.9, 3.2)	.001	1.6 (0.4, 2.8)	.008
	Panic	0.8 (0.5, 1.2)	<.001	0.7 (0.3, 1.1)	<.001
	Generalized anxiety	0.2 (−0.1, 0.6)	.219	0.2 (−0.2, 0.6)	.321
	Separation anxiety	0.5 (0.2, 0.8)	.001	0.4 (0.1, 0.7)	.007
	Social anxiety	0.3 (−0.01, 0.6)	.058	0.2 (−0.1, 0.5)	.246
	School avoidance	0.2 (0.01, 0.3)	.043	0.1 (−0.03, 0.3)	.127

PBDE-99	Total score	2.1 (0.9, 3.2)	<.001	1.7 (0.5, 2.9)	.004
	Panic	0.8 (0.4, 1.2)	<.001	0.7 (0.3, 1.1)	.000
	Generalized anxiety	0.3 (−0.1, 0.6)	.144	0.2 (−0.1, 0.6)	.212
	Separation anxiety	0.5 (0.3, 0.8)	<.001	0.4 (0.1, 0.7)	.003
	Social anxiety	0.3 (−0.02, 0.6)	.065	0.2 (−0.1, 0.5)	.262
	School avoidance	0.2 (0.03, 0.3)	.018	0.2 (−0.01, 0.3)	.062

PBDE-100	Total score	1.4 (0.2, 2.5)	.021	0.9 (−0.2, 2)	.116
	Panic	0.6 (0.2, 0.9)	.003	0.4 (0.1, 0.8)	.022
	Generalized anxiety	0.1 (−0.2, 0.5)	.493	0.1 (−0.3, 0.4)	.701
	Separation anxiety	0.4 (0.1, 0.6)	.016	0.2 (−0.04, 0.5)	.089
	Social anxiety	0.2 (−0.1, 0.5)	.226	0.1 (−0.2, 0.4)	.676
	School avoidance	0.1 (−0.03, 0.3)	.113	0.1 (−0.1, 0.2)	.269

PBDE-153	Total score	0.7 (−0.5, 1.8)	.251	0.4 (−0.7, 1.5)	.451
	Panic	0.2 (−0.1, 0.6)	.216	0.2 (−0.2, 0.5)	.364
	Generalized anxiety	0.1 (−0.3, 0.4)	.696	0.03 (−0.3, 0.4)	.855
	Separation anxiety	0.1 (−0.2, 0.4)	.379	0.1 (−0.2, 0.3)	.587
	Social anxiety	0.2 (−0.1, 0.5)	.251	0.1 (−0.2, 0.4)	.489
	School avoidance	0.1 (−0.1, 0.2)	.469	0.04 (−0.1, 0.2)	.592

∑PBDE	Total score	2.1 (0.8, 3.4)	.002	1.6 (0.3, 2.9)	.019
	Panic	0.7 (0.3, 1.2)	.001	0.6 (0.2, 1)	.007
	Generalized anxiety	0.3 (−0.04, 0.7)	.081	0.3 (−0.1, 0.7)	.191
	Separation anxiety	0.5 (0.2, 0.9)	.003	0.4 (0.04, 0.7)	.027
	Social anxiety	0.4 (−0.002, 0.7)	.053	0.2 (−0.1, 0.6)	.216
	School avoidance	0.2 (−0.02, 0.3)	.081	0.1 (−0.1, 0.3)	.228

Abbreviation: PBDE, polybrominated diphenyl ether.

a Adjusted for sex, maternal age, marital status, education, income at 12-year visit, maternal Symptom Checklist-90-R anxiety score, and relational frustration score.

**TABLE 3:** Relative risk for elevated^a^ Child Anxiety and Related Disorders (SCARED) score by each doubling of maternal serum PBDE concentration (ng/g lipid)

		Unadjusted		Adjusted^b^	
Congener	SCARED score	RR (95% CI) for log_2_ PBDE concentration	*p* Value	RR (95% CI) for log_2_ PBDE concentration	*p* Value
PBDE-28	Total score	1.31 (1.16, 1.48)	<.001	1.26 (1.11, 1.42)	<.001
	Panic	1.35 (1.16, 1.57)	<.001	1.3 (1.13, 1.51)	<.001
	Generalized anxiety	1 (0.8, 1.27)	.969	0.99 (0.79, 1.25)	.945
	Separation anxiety	1.2 (1.06, 1.35)	.003	1.16 (1.03, 1.3)	.015
	Social anxiety	1.18 (0.99, 1.41)	.060	1.11 (0.95, 1.31)	.187
	School avoidance	1.14 (0.96, 1.36)	.125	1.13 (0.95, 1.34)	.172

PBDE-47	Total score	1.25 (1.13, 1.39)	<.001	1.2 (1.07, 1.33)	.001
	Panic	1.33 (1.19, 1.5)	<.001	1.3 (1.15, 1.48)	<.001
	Generalized anxiety	0.99 (0.81, 1.2)	.894	0.99 (0.81, 1.21)	.953
	Separation anxiety	1.19 (1.07, 1.33)	.001	1.13 (1.02, 1.26)	.016
	Social anxiety	1.15 (0.99, 1.34)	.060	1.06 (0.92, 1.22)	.419
	School avoidance	1.1 (0.95, 1.27)	.217	1.07 (0.92, 1.26)	.369

PBDE-99	Total score	1.23 (1.12, 1.36)	<.001	1.18 (1.07, 1.3)	.001
	Panic	1.28 (1.14, 1.44)	<.001	1.25 (1.11, 1.41)	<.001
	Generalized anxiety	0.97 (0.81, 1.17)	.776	0.99 (0.82, 1.19)	.891
	Separation anxiety	1.21 (1.1, 1.33)	<.001	1.15 (1.04, 1.26)	.006
	Social anxiety	1.14 (0.98, 1.32)	.099	1.05 (0.91, 1.21)	.517
	School avoidance	1.15 (1, 1.31)	.048	1.12 (0.97, 1.3)	.112

PBDE-100	Total score	1.19 (1.07, 1.32)	.001	1.13 (1.01, 1.26)	.027
	Panic	1.2 (1.05, 1.36)	.005	1.16 (1.02, 1.31)	.023
	Generalized anxiety	0.99 (0.82, 1.21)	.946	0.99 (0.8, 1.22)	.927
	Separation anxiety	1.12 (1.01, 1.25)	.035	1.08 (0.97, 1.2)	.182
	Social anxiety	1.08 (0.93, 1.27)	.301	1 (0.86, 1.17)	.987
	School avoidance	1.08 (0.94, 1.24)	.284	1.06 (0.92, 1.23)	.410

PBDE-153	Total score	1.13 (1.01, 1.25)	.028	1.11 (0.987, 1.24)	.082
	Panic	1.09 (0.95, 1.25)	.207	1.07 (0.93, 1.23)	.337
	Generalized anxiety	1.08 (0.88, 1.33)	.442	1.08 (0.88, 1.33)	.461
	Separation anxiety	1.01 (0.89, 1.14)	.905	0.99 (0.87, 1.11)	.833
	Social anxiety	1.01 (0.85, 1.2)	.900	0.97 (0.81, 1.15)	.698
	School avoidance	1.02 (0.88, 1.18)	.786	1.01 (0.89, 1.15)	.861

∑PBDE	Total score	1.26 (1.13, 1.41)	<.001	1.19 (1.06, 1.33)	.002
	Panic	1.29 (1.13, 1.48)	<.001	1.24 (1.08, 1.42)	.002
	Generalized anxiety	1.01 (0.82, 1.26)	.903	1 (0.8, 1.26)	.980
	Separation anxiety	1.19 (1.07, 1.32)	.001	1.12 (1.01, 1.25)	.027
	Social anxiety	1.13 (0.95, 1.33)	.166	1.04 (0.89, 1.23)	.607
	School avoidance	1.08 (0.91, 1.27)	.377	1.04 (0.88, 1.22)	.641

Abbreviation: PBDE, polybrominated diphenyl ether.

a Score above clinically relevant cutoff: total (≥25), panic/somatic (≥7), generalized anxiety (≥9), separation anxiety (≥5), social anxiety (≥8), school avoidance (≥3).

b Adjusted for sex, maternal age, marital status, education, income at 12-year visit, maternal Symptom Checklist-90-R anxiety score, and relational frustration score.

**TABLE 4:** Difference in Children’s Depression Inventory (CDI) *T*-scores by each doubling of maternal serum PBDE concentration (ng/g lipid)

		Unadjusted		Adjusted^a^	
Congener	CDI scale/subscale	*β* estimate (95% CI) for log_2_ PBDE concentration	*p* Value	*β* estimate (95% CI) for log_2_ PBDE concentration	*p* Value
PBDE-28	*T*-score	1.8 (0.6, 2.9)	.004	1.5 (0.4, 2.6)	.007
	Emotional problems	1.5 (0.3, 2.6)	.011	1.2 (0.2, 2.3)	.021
	Negative mood	1.6 (0.3, 2.8)	.014	1.3 (0.2, 2.5)	.028
	Negative self-esteem	1 (−0.04, 2)	.061	0.8 (−0.1, 1.8)	.093
	Functional problems	1.8 (0.6, 3)	.003	1.6 (0.5, 2.7)	.006
	Ineffectiveness	2 (0.8, 3.2)	.001	1.8 (0.7, 2.9)	.002
	Interpersonal problems	0.9 (−0.2, 2.1)	.115	0.8 (−0.4, 1.9)	.178

PBDE-47	*T*-score	1.1 (0.2, 2.1)	.024	0.8 (−0.1, 1.8)	.082
	Emotional problems	1 (0.04, 1.9)	.042	0.8 (−0.1, 1.7)	.100
	Negative mood	1.1 (0.1, 2.1)	.040	0.8 (−0.2, 1.8)	.127
	Negative self-esteem	0.6 (−0.3, 1.4)	.190	0.5 (−0.3, 1.4)	.212
	Functional problems	1.1 (0.1, 2.1)	.036	0.8 (−0.2, 1.8)	.130
	Ineffectiveness	1 (0.05, 2)	.041	0.8 (−0.2, 1.8)	.127
	Interpersonal problems	0.9 (−0.1, 1.8)	.079	0.6 (−0.4, 1.5)	.254

PBDE-99	*T*-score	1.2 (0.2, 2.1)	.015	0.9 (−0.03, 1.9)	.059
	Emotional problems	1 (0.1, 1.9)	.025	0.9 (−0.03, 1.8)	.059
	Negative mood	1.3 (0.3, 2.3)	.011	1.1 (0.1, 2.1)	.038
	Negative self-esteem	0.4 (−0.4, 1.2)	.385	0.3 (−0.5, 1.2)	.432
	Functional problems	1.1 (0.1, 2)	.031	0.8 (−0.2, 1.7)	.132
	Ineffectiveness	0.9 (−0.03, 1.9)	.058	0.7 (−0.3, 1.7)	.184
	Interpersonal problems	1 (0.1, 1.9)	.036	0.7 (−0.3, 1.6)	.159

PBDE-100	*T*-score	0.8 (−0.1, 1.8)	.099	0.6 (−0.3, 1.5)	.185
	Emotional problems	0.7 (−0.2, 1.6)	.140	0.6 (−0.3, 1.5)	.204
	Negative mood	0.8 (−0.2, 1.8)	.106	0.6 (−0.3, 1.6)	.196
	Negative self-esteem	0.3 (−0.5, 1.1)	.507	0.3 (−0.5, 1.1)	.481
	Functional problems	0.7 (−0.2, 1.7)	.140	0.5 (−0.4, 1.5)	.287
	Ineffectiveness	0.7 (−0.3, 1.7)	.167	0.5 (−0.5, 1.5)	.302
	Interpersonal problems	0.6 (−0.3, 1.6)	.179	0.4 (−0.5, 1.3)	.373

PBDE-153	*T*-score	0.2 (−0.7, 1.2)	.656	0.2 (−0.7, 1.1)	.620
	Emotional problems	0.3 (−0.6, 1.2)	.487	0.3 (−0.5, 1.2)	.445
	Negative mood	0.6 (−0.4, 1.6)	.274	0.6 (−0.4, 1.5)	.251
	Negative self-esteem	−0.2 (−1, 0.6)	.704	−0.1 (−0.9, 0.7)	.760
	Functional problems	0.04 (−0.9, 1)	.939	0.04 (−0.9, 1)	.927
	Ineffectiveness	0.04 (−0.9, 1)	.932	0.04 (−0.9, 1)	.936
	Interpersonal problems	0.04 (−0.9, 1)	.932	0.1 (−0.8, 0.9)	.895

∑PBDE	*T*-score	1.1 (0.1, 2.2)	.038	0.8 (−0.2, 1.8)	.109
	Emotional problems	0.9 (−0.1, 1.9)	.078	0.7 (−0.3, 1.6)	.175
	Negative mood	1.1 (−0.1, 2.2)	.065	0.8 (−0.3, 1.9)	.155
	Negative self-esteem	0.5 (−0.5, 1.4)	.326	0.3 (−0.6, 1.2)	.497
	Functional problems	1.2 (0.1, 2.3)	.036	0.9 (−0.2, 1.9)	.109
	Ineffectiveness	1.2 (0.1, 2.3)	.031	0.9 (−0.1, 2)	.091
	Interpersonal problems	0.8 (−0.3, 1.8)	.155	0.5 (−0.5, 1.6)	.329

Abbreviation: PBDE, polybrominated diphenyl ether.

a Adjusted for maternal age, marital status, education, income at 12-year visit, relational frustration score, and maternal Symptom Checklist-90-R depression score.

## Data Availability

The data that support the findings of this study are available on request from the corresponding author. The data are not publicly available due to privacy or ethical restrictions.
