# Supplementary material for: Early exposure to flame retardants is prospectively associated with anxiety symptoms in adolescents: A prospective birth cohort study
Source: Depress Anxiety. Author manuscript; Available in PMC 2023 Apr 17. (PMC10092502; doi:10.1002/da.23284)
Supplement: Supplemental [file NIHMS1887396-supplement-Supplemental.docx]

**Supplemental Table 1: Selected prior associations between PBDE and pediatric mental health or developmental outcomes**

| **Source/timing of PBDE measure** | **Sample age** | **Sample origin** | **Mental health and developmental outcomes** | **Reference** |
| --- | --- | --- | --- | --- |
| Cord blood | 24 months | China | 10-fold increase in PBDE-99 is associated with a 2-point decrease in language domain quotient and a 10-fold increase in PBDE-47 was associated with a 2-point decrease in social domain quotient | Ding et al. 2015 |
| Maternal prenatal blood (early pregnancy) |  | Canada | 10-fold increase in maternal blood PBDE concentration associated with lower full-scale intelligence quotient (IQ) in boys (-3.4).  PBDE-related decreases in verbal and performance IQ | Azar et al. 2021 |
| Maternal prenatal, blood | 5 and  7 years | United States (California) | Maternal prenatal PBDE associated with impaired attention at 5 and 7 years. | Eskenazi et al. 2013 |
| Maternal prenatal blood and child samples collected at 1, 2, 3, 5 and 8 years | 8 years | United States (Midwest) | A 10-fold increase in concurrent concentrations of PBDE was associated with deficits in behavioral regulation. PBDEs at 8 years. | Vuong et al. 2018 |
| Cord blood | 4 years  6 years | United Sates (Northeast) | Higher PBDE concentrations associated with increased attention problems ate age 4 but not at age 6. | Cowell et al. 2015 |
| Cord blood,  Child blood (age 9 years) | 9 years,  10 ½ years and  12 years | United States (California) | Prenatal PBDE associated with decreased executive function at ages 9 and 12, including poorer working memory and measures of attention and executive function. | Sagiv et al. 2015 |
| Maternal prenatal, blood | 8 years | United States (Midwest) | Higher prenatal PBDE concentrations associated with lower reading skills and full-scale IQ and more externalizing behaviors at 8 years of age. | Zang et al. 2017 |

**Supplemental Table 2: Maternal serum PBDE concentrations (ng/g lipid).**

| **Congener** | **N** | **Min** | **25th %ile** | **Median** | **75th %ile** | **90th %ile** | **Max** | **Geometric Mean (95%CI)** | **%>LOD** |
| --- | --- | --- | --- | --- | --- | --- | --- | --- | --- |
| PBDE-28 | 200 | 0.3 | 0.6 | 1.0 | 1.6 | 3.4 | 16.7 | 1.1 (1 - 1.2) | 87.0 |
| PBDE-47 | 236 | 2.2 | 11.0 | 20.0 | 34.6 | 63.5 | 539.0 | 20.8 (18.4 - 23.4) | 99.2 |
| PBDE-99 | 235 | 0.6 | 2.5 | 4.5 | 7.9 | 16.6 | 193.0 | 4.7 (4.2 - 5.4) | 99.1 |
| PBDE-100 | 234 | 0.4 | 2.0 | 3.5 | 6.7 | 13 | 107.0 | 3.9 (3.4 - 4.4) | 95.7 |
| PBDE-153 | 236 | 0.6 | 2.4 | 4.2 | 8.2 | 20.5 | 152.0 | 4.8 (4.2 - 5.4) | 95.8 |
| ∑PBDE | 200 | 5.2 | 19.2 | 34.1 | 66.0 | 113.0 | 905.8 | 37.1 (32.7 - 42.1) | 100.0 |

| **Supplemental Table 3: Adjusted* difference in scores of Screen for Child Anxiety and Related Disorders (SCARED) by each doubling of maternal serum PBDE concentration (ng/g lipid) from secondary analysis adjusting for maternal blood mercury and lead concentrations.** | | | |
| --- | --- | --- | --- |
| **Congener** | **SCARED Score** | **Estimate (95%CI) for for log_2_ PBDE concentration** | **p-value** |
| PBDE-28 | total score | 2.1 (0.7, 3.5) | 0.004 |
|  | panic | 0.7 (0.3, 1.2) | 0.002 |
|  | generalized anxiety | 0.3 (-0.1, 0.8) | 0.139 |
|  | separation anxiety | 0.4 (0.1, 0.8) | 0.017 |
|  | social anxiety | 0.4 (-0.02, 0.7) | 0.064 |
|  | school avoidance | 0.2 (0.01, 0.4) | 0.037 |
| PBDE-47 | total score | 1.6 (0.4, 2.8) | 0.008 |
|  | panic | 0.7 (0.3, 1.1) | 0.000 |
|  | generalized anxiety | 0.2 (-0.2, 0.6) | 0.338 |
|  | separation anxiety | 0.4 (0.1, 0.7) | 0.007 |
|  | social anxiety | 0.2 (-0.1, 0.5) | 0.236 |
|  | school avoidance | 0.1 (-0.04, 0.3) | 0.154 |
| PBDE-99 | total score | 1.7 (0.5, 2.9) | 0.005 |
|  | panic | 0.7 (0.3, 1.1) | 0.001 |
|  | generalized anxiety | 0.2 (-0.1, 0.6) | 0.231 |
|  | separation anxiety | 0.4 (0.1, 0.7) | 0.003 |
|  | social anxiety | 0.2 (-0.1, 0.5) | 0.247 |
|  | school avoidance | 0.1 (-0.02, 0.3) | 0.083 |
| PBDE-100 | total score | 0.9 (-0.3, 2) | 0.131 |
|  | panic | 0.5 (0.1, 0.8) | 0.022 |
|  | generalized anxiety | 0.05 (-0.3, 0.4) | 0.786 |
|  | separation anxiety | 0.2 (-0.04, 0.5) | 0.089 |
|  | social anxiety | 0.1 (-0.2, 0.4) | 0.683 |
|  | school avoidance | 0.1 (-0.1, 0.2) | 0.344 |
| PBDE-153 | total score | 0.4 (-0.7, 1.5) | 0.483 |
|  | panic | 0.2 (-0.2, 0.6) | 0.355 |
|  | generalized anxiety | 0.01 (-0.3, 0.4) | 0.933 |
|  | separation anxiety | 0.1 (-0.2, 0.3) | 0.595 |
|  | social anxiety | 0.1 (-0.2, 0.4) | 0.525 |
|  | school avoidance | 0.04 (-0.1, 0.2) | 0.638 |
| ∑PBDE | total score | 1.6 (0.3, 2.9) | 0.020 |
|  | panic | 0.6 (0.2, 1) | 0.008 |
|  | generalized anxiety | 0.3 (-0.1, 0.7) | 0.201 |
|  | separation anxiety | 0.4 (0.05, 0.7) | 0.026 |
|  | social anxiety | 0.2 (-0.1, 0.6) | 0.200 |
|  | school avoidance | 0.1 (-0.1, 0.3) | 0.267 |
|  |  |  |  |
| *Adjusted for child sex, maternal age, marital status, education, income at 12-year visit, maternal Symptom Checklist-90-R anxiety score, and relational frustration score, maternal blood lead and maternal blood mercury. | | | |

**Supplemental Table 4: Sex-specific adjusted* difference in scores of Screen for Child Anxiety and Related Disorders (SCARED) and Children’s Depression Inventory (CDI) T-scores by each doubling of maternal serum PBDE concentration (ng/g lipid).**

|  |  | **Females (n=132)** |  | **Males (n=104)** |  |  |
| --- | --- | --- | --- | --- | --- | --- |
| **Congener** | **Score** | **β estimate (95%CI) for log_2_ PBDE concentration** | **p-value** | **β estimate (95%CI) for log_2_ PBDE concentration** | **p-value** | **p-value of PBDE by sex interaction** |
| PBDE-28 | SCARED total score | 1.3 (-0.4 , 3.0) | 0.138 | 3.6 (1.2 , 6.0) | 0.003 | 0.117 |
| PBDE-47 | SCARED total score | 1.0 (-0.4 , 2.4) | 0.170 | 2.9 (0.9 , 5.0) | 0.005 | 0.115 |
| PBDE-99 | SCARED total score | 1.5 (0.1 , 2.9) | 0.035 | 2.1 (0.1 , 4.1) | 0.037 | 0.620 |
| PBDE-100 | SCARED total score | 0.3 (-1.1 , 1.7) | 0.646 | 2.0 (0.1 , 3.9) | 0.037 | 0.154 |
| PBDE-153 | SCARED total score | -0.1 (-1.6 , 1.3) | 0.862 | 1.2 (-0.5 , 2.9) | 0.168 | 0.242 |
| Σ PBDE | SCARED total score | 0.9 (-0.7 , 2.5) | 0.251 | 2.8 (0.6 , 5.0) | 0.012 | 0.170 |
| PBDE-28 | CDI total score | 1.1 (-0.2 , 2.4) | 0.099 | 2.2 (0.4 , 4.1) | 0.020 | 0.334 |
| PBDE-47 | CDI total score | 0.8 (-0.3 , 2.0) | 0.152 | 0.7 (-0.9 , 2.4) | 0.389 | 0.909 |
| PBDE-99 | CDI total score | 1.0 (-0.2 , 2.1) | 0.093 | 0.7 (-0.9 , 2.3) | 0.386 | 0.785 |
| PBDE-100 | CDI total score | 0.6 (-0.5 , 1.7) | 0.291 | 0.5 (-1.1 , 2.0) | 0.566 | 0.870 |
| PBDE-153 | CDI total score | 0.7 (-0.5 , 1.9) | 0.231 | 0.1 (-1.2 , 1.5) | 0.840 | 0.947 |
| Σ PBDE | CDI total score | 0.6 (-0.6 , 1.9) | 0.328 | 1.1 (-0.6 , 2.8) | 0.217 | 0.663 |

* Estimates for SCARED scores adjusted for maternal age, marital status, education, income at 12-year visit, maternal Symptom Checklist-90-R anxiety score, and relational frustration score; Estimates for CDI scores adjusted for maternal age, marital status, education, income at 12-year visit, maternal Symptom Checklist-90-R depression score, and relational frustration score.

**Supplemental Figure 1.** Directed acyclic graph illustrating the causal diagram for the current study


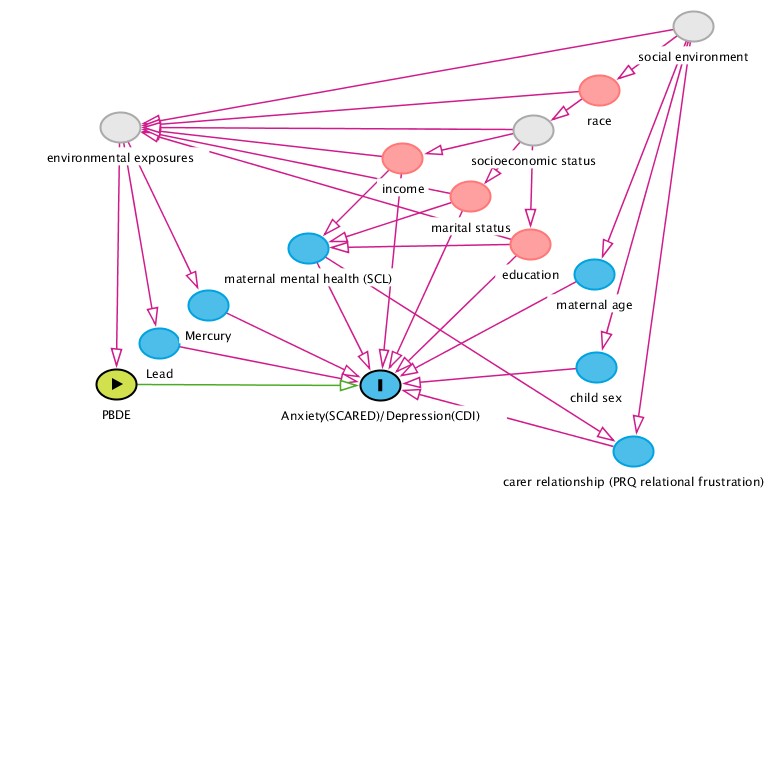


Legend:


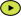
 exposure


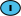
 outcome


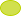
 ancestor of exposure


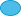
 ancestor of outcome


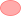
 ancestor of exposure *and* outcome


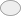
 unobserved (latent)


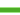
 causal path


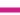
 biasing path

# For Maternal mental health, SCL-90 anxiety score will be used for the analysis of the anxiety (SCARED) outcomes, while SCL-90 depression score will be used for the analysis of the depression (CDI) symptoms.
